# Small ubiquitin-related modifier 1 is involved in hepatocellular carcinoma progression via mediating p65 nuclear translocation

**DOI:** 10.18632/oncotarget.8066

**Published:** 2016-03-14

**Authors:** Jun Liu, Xiaofang Tao, Jin Zhang, Peng Wang, Manqi Sha, Yong Ma, Xiaoping Geng, Lijie Feng, Yujun Shen, Yifan Yu, Siying Wang, Shengyun Fang, Yuxian Shen

**Affiliations:** ^1^ School of Basic Medical Sciences, Anhui Medical University, Hefei, China; ^2^ School of Pharmacy, Anhui Medical University, Hefei, China; ^3^ Biopharmaceutical Research Institute, Anhui Medical University, Hefei, China; ^4^ Chinese People's Liberation Army 123 Hospital, Bengbu, China; ^5^ The First Affiliated Hospital, Anhui Medical University, Hefei, China; ^6^ Actuarial Science, School of Continuing Education, Columbia University, New York, NY, USA; ^7^ Center for Biomedical Engineering and Technology, Department of Physiology, University of Maryland School of Medicine, Baltimore, MD, USA

**Keywords:** SUMO1, SUMOylation, hepatocellular carcinoma, p65, NF-κB

## Abstract

Small ubiquitin-related modifier (SUMO) proteins participate in a post-translational modification called SUMOylation and regulate a variety of intracellular processes, such as targeting proteins for nuclear import. The nuclear transport of p65 results in the activation of NF-κB, and p65 contains several SUMO interacting motifs (SIMs). However, the relationship between p65 and SUMO1 in hepatocellular carcinoma (HCC) remains unclear. In this study, we demonstrated the potential roles of SUMO1 in HCC via the regulation of p65 subcellular localization. We found that either SUMO1- or p65-positive immunoreactivity was remarkably increased in the nuclei of tumor tissues in HCC patients compared with non-tumor tissues, and further analysis suggested a correlation between SUMO1- and nuclear p65-positive immunoreactivities (R = 0.851, *P* = 0.002). We also verified the interaction between p65 and SUMO1 in HCC by co-immunoprecipitation. TNF-α and hypoxia increased SUMO1 protein levels and enhanced SUMO1-modified p65 SUMOylation. Moreover, the knockdown of SUMO1 decreased p65 nuclear translocation and inhibited NF-κB transcriptional activity. Further the results of this study revealed that the knockdown of SUMO1 suppressed the proliferation and migration of hepatoma cells. These results suggest that SUMO1 contributes to HCC progression by promoting p65 nuclear translocation and regulating NF-κB activity.

## INTRODUCTION

Small ubiquitin-related modifier 1 (SUMO1) protein-mediated SUMOylation is involved in various cellular processes, including targeting proteins for nuclear import [1, 2]. A previous study demonstrated that SUMO1 is up-regulated in human liver tumor tissues and hepatoma cell lines and that the knockdown of SUMO1 leads to an increase in the S to G2 phase ratio and the inhibition of SMMC7721 cell proliferation [3]. SUMO1-modified centrosomal protein P4.1-associated protein (CPAP) SUMOylation increases NF-κB activity by collaborating with HBx proteins in HBV-related hepatocellular carcinomas (HCCs) [4]. SUMO1-related Shp2 SUMOylation activates ERK and promotes the development of HCC [5]. Additionally, SUMO1-modified SUMOylation is associated with multidrug resistance in HCC [6]. These results suggest that SUMO1-related SUMOylation contributes to hepatocarcinogenesis and HCC progression.

The NF-κB pathway is associated with the development of HCC [7–9]. Nuclear p65 is an activated form of NF-κB that is up-regulated in the liver tumor tissues and is associated with hepatocarcinogenesis [10]. Additionally, p65 contains several SUMO interacting motifs (SIMs) and can be SUMOylated by SUMO3 [11]. Moreover, we recently reported that SUMO2/3 can interact with p65, and SUMO2/3-mediated p65 SUMOylation promotes the stability of cytoplasmic p65 by antagonizing ubiquitination-mediated p65 degradation in the hepatoma cells [12]. However, the relationship between SUMO1 and p65 in HCC remains unclear.

In this study, we investigated SUMO1 and p65 expressions in human liver samples and observed a close correlation. Next, we verified the interaction of SUMO1-p65 and found that SUMO1 mediated p65 SUMOylation. Furthermore, SUMO1-modified p65 SUMOylation was enhanced by oxygen and glucose deprivation (OGD) or TNF-α treatment. Moreover, the knockdown of SUMO1 decreased hypoxia or TNF-α-induced p65 nuclear translocation and inhibited NF-κB activation. Additionally, we observed suppressive effects of SUMO1 knockdown on the proliferation and migration of hepatoma cells *in vitro*.

## RESULTS

### Correlation between SUMO1 and NK-κB p65 in human liver tissues

**Figure 1:**
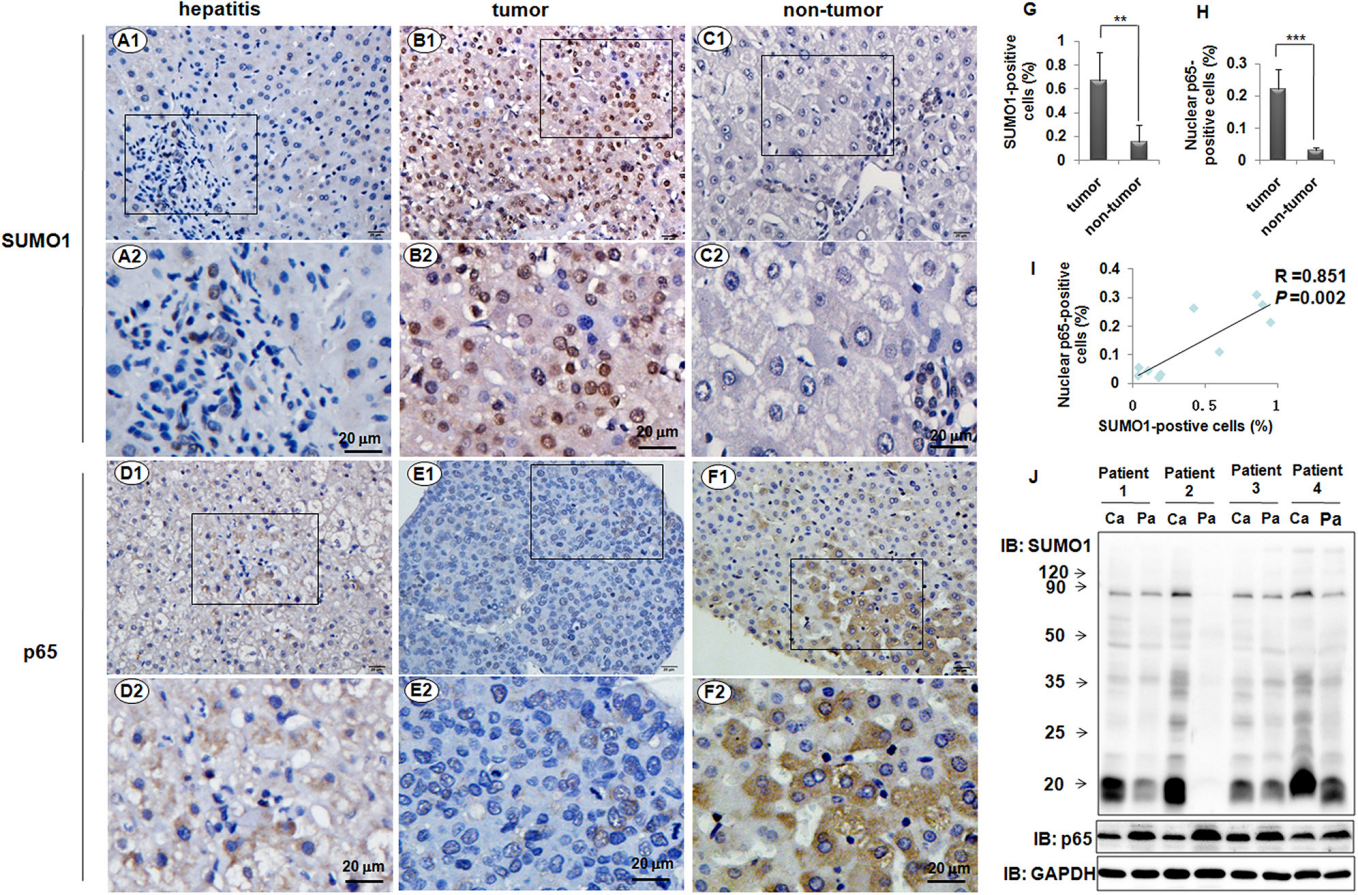
Expressions of SUMO1 and p65 in liver tissues SUMO1 was detected in the human liver tissues from the patients with hepatitis B (**A1**-2), HCC (**B1**-2), and the corresponding adjacent non-tumor (**C1**-2) by using immunohistochemistry assays. Immunohistochemistry was also performed to detect p65 in the liver tissues from the patients with hepatitis B (**D1**-2), HCC (**E1**-2), and the corresponding adjacent non-tumor (**F1**-2). The images of the rectangles in A1–F1 are magnified in A2–F2. Scale bar = 20 μm. The SUMO1-positive cells (**G**) and nuclear p65-positive cells (**H**) were analyzed in the tumor and corresponding non-tumor liver tissues from the same patients (*n* = 8), and the percentages were calculated. **P < 0.01, ***P <0.001, compared with non-tumor. (**I**) Correlation between the percentages of SUMO1-positive and nuclear p65-positive cells. R = 0.851, *P* = 0.002, *n* = 10. (**J**) The levels of SUMO1 and p65 were detected by western blot in the tumor and non-tumor liver tissues of the HCC patients. Ca: tumor; Pa: para-tumor.

To observe the expression characteristics of SUMO1 protein and p65 in human liver tissues, we used immunohistochemistry assays. The liver tissues from 5 hepatitis patients and 8 HCC patients were used. The results revealed that only a few cells were SUMO1-positive in the tissues of the hepatitis B patients, and SUMO1 was mainly localized in the nuclei (Figure 1A). We observed p65-positive cells in the tissues of the hepatitis B patients, and p65 was mainly localized in the cytoplasm (Figure 1D). Both nuclear SUMO1 and nuclear p65 in the tumor tissues were remarkably increased compared with the levels in the adjacent non-tumor tissues (*P* < 0.01 and *P* < 0.001, respectively, Figure 1B–1C, 1E–1F and 1G–1H). Additionally, we found a close correlation between SUMO1 and nuclear p65 in the liver tissues of HCC patients with HBV infections (R = 0.851, *P* = 0.002, Figure 1I). However, the total levels of SUMO1 and p65 were increased in the tumor tissues and the adjacent non-tumor tissues, respectively (Figure 1J).

### SUMO1 interacts with p65

**Figure 2:**
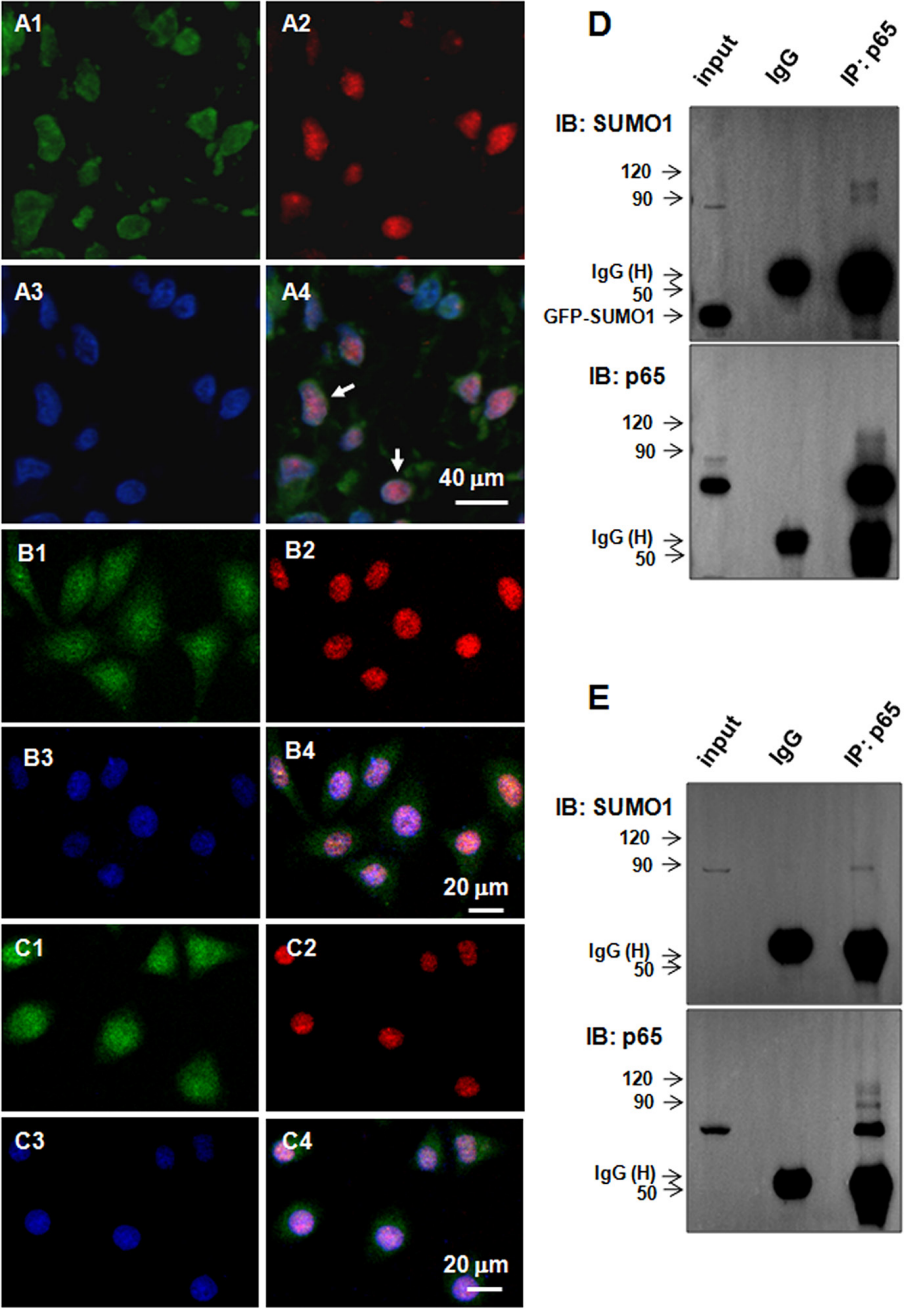
SUMO1 interacts with p65 in the liver tumor tissues and hepatoma cells (**A**–**C**) Co-localization of SUMO1 and p65. Immunofluorescent staining was performed using antibodies against p65 (green) and SUMO1 (red) in the tumor tissues of HCC patients (**A1**-4), HepG2 cells (**B1**-4), and SMMC7721 cells (**C1**-4). The nuclei were stained with DAPI (blue). The scale bars are indicated. (**D**–**E**) SUMO1 protein interacts with p65. (D) SMMC7721 cells were harvested for co-immunoprecipitation assays 24 hours after transfection with GFP-SUMO1 and myc-UBC9. The isotype IgG was used as a negative control, and 2% total lysate was loaded as input. (E) The tumor tissues of the HCC patients were lysed to process the co-immunoprecipitation assays using anti-p65 antibody. The isotype IgG was used as a negative control, and 1% total lysate was loaded as input.

Immunofluorescence staining revealed SUMO1 and p65 co-localized in the nuclei in the HCC tissues (Figure 2A, as indicated by the arrows) and hepatoma cells (Figure 2B–2C). We performed co-immunoprecipitation assays to verify the interaction of SUMO1 and p65. SMMC7721 cells were co-transfected with myc-Ubc9 and GFP-SUMO1, which can enhance the SUMO1-related SUMOylation [13]. We found that high molecular weight bands (approximately 80 kD–120 kD) were detected by anti-SUMO1 antibody following co-immunoprecipitation with anti-p65 antibody (Figure 2D, upper panel). Moreover, high molecular weight bands were found in the HCC tissues using anti-SUMO1 antibody following co-immunoprecipitation with anti-p65 antibody (Figure 2E, upper panel). Additionally, high molecular weight bands were detected after blotting p65 with p65 antibody (Figure 2D–2E, lower panel). These results suggest that SUMO1 interacts with p65 and promotes p65 SUMOylation.

### TNF-α and OGD treatments up-regulate SUMO1 protein levels

**Figure 3:**
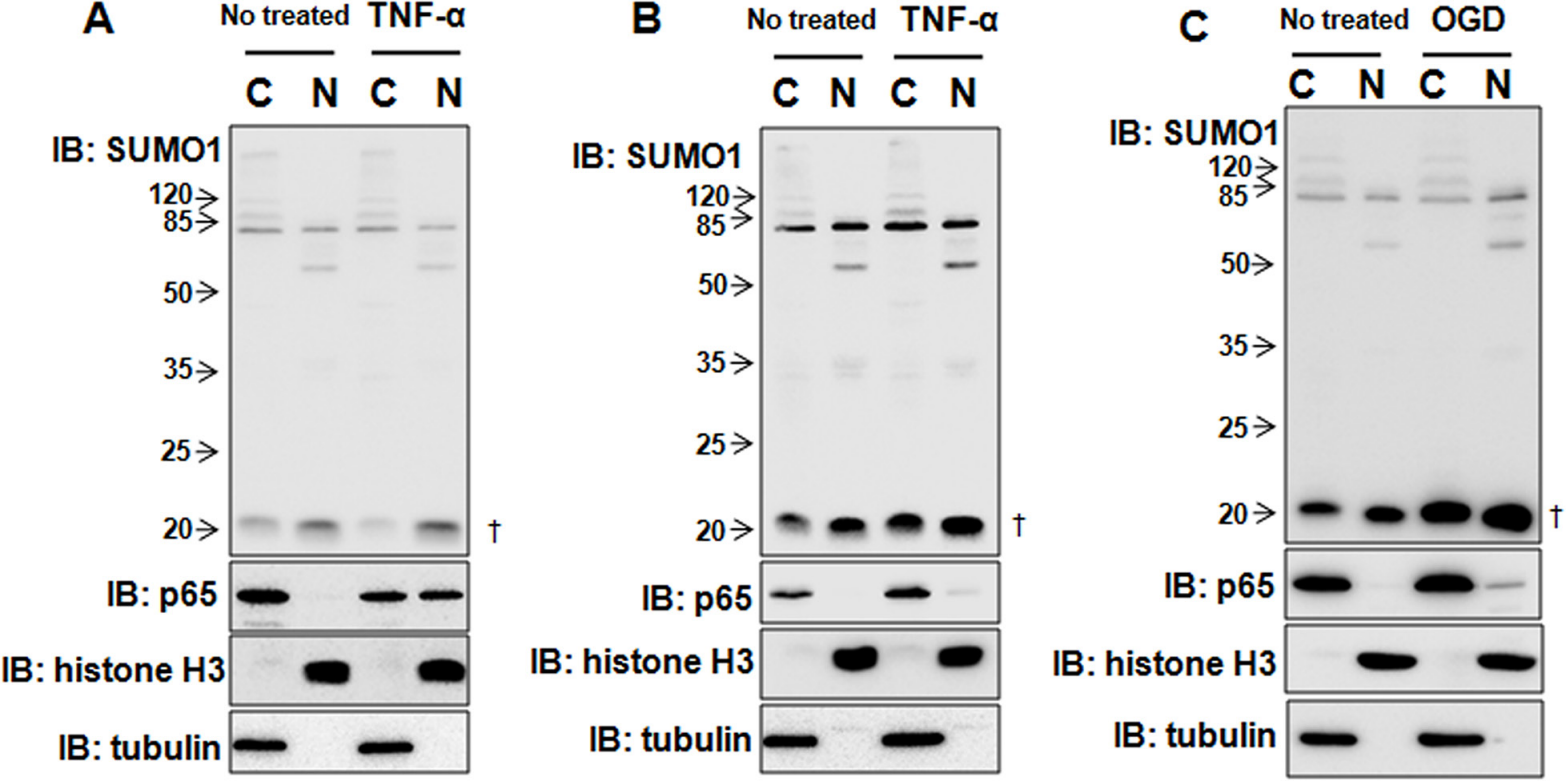
TNF-α and hypoxia differentially regulate SUMO1 expression SMMC7721 cells were cultured with 10 ng/ml TNF-α for 30 min (**A**) or 8 hours (**B**) or treated with OGD for 150 min (**C**). Then, the nuclear and cytoplasmic proteins were extracted and processed for western blot. Tubulin and histone H3 were used as the cytoplasmic and nuclear markers. C: cytoplasm; N: nucleus. ^†^: free SUMO1.

Due to the chronic inflammatory response, hypoxia and inadequate energy supplies occur in the tumor tissues of HCC patients [14]. Therefore, we wondered whether inflammation or hypoxia affected SUMO1 expression. The results revealed that 10 ng/ml TNF-α treatment for 30 min had no effect on SUMO1 expression (Figure 3A), whereas TNF-α treatment for 8 hours remarkably increased SUMO1 levels (Figure 3B). Moreover, OGD treatment for 150 min also increased SUMO1 expression (Figure 3C). These findings suggest that hypoxia and inflammation increase SUMO1 protein levels.

### TNF-α and OGD treatments enhance the interaction between SUMO1 and p65

**Figure 4:**
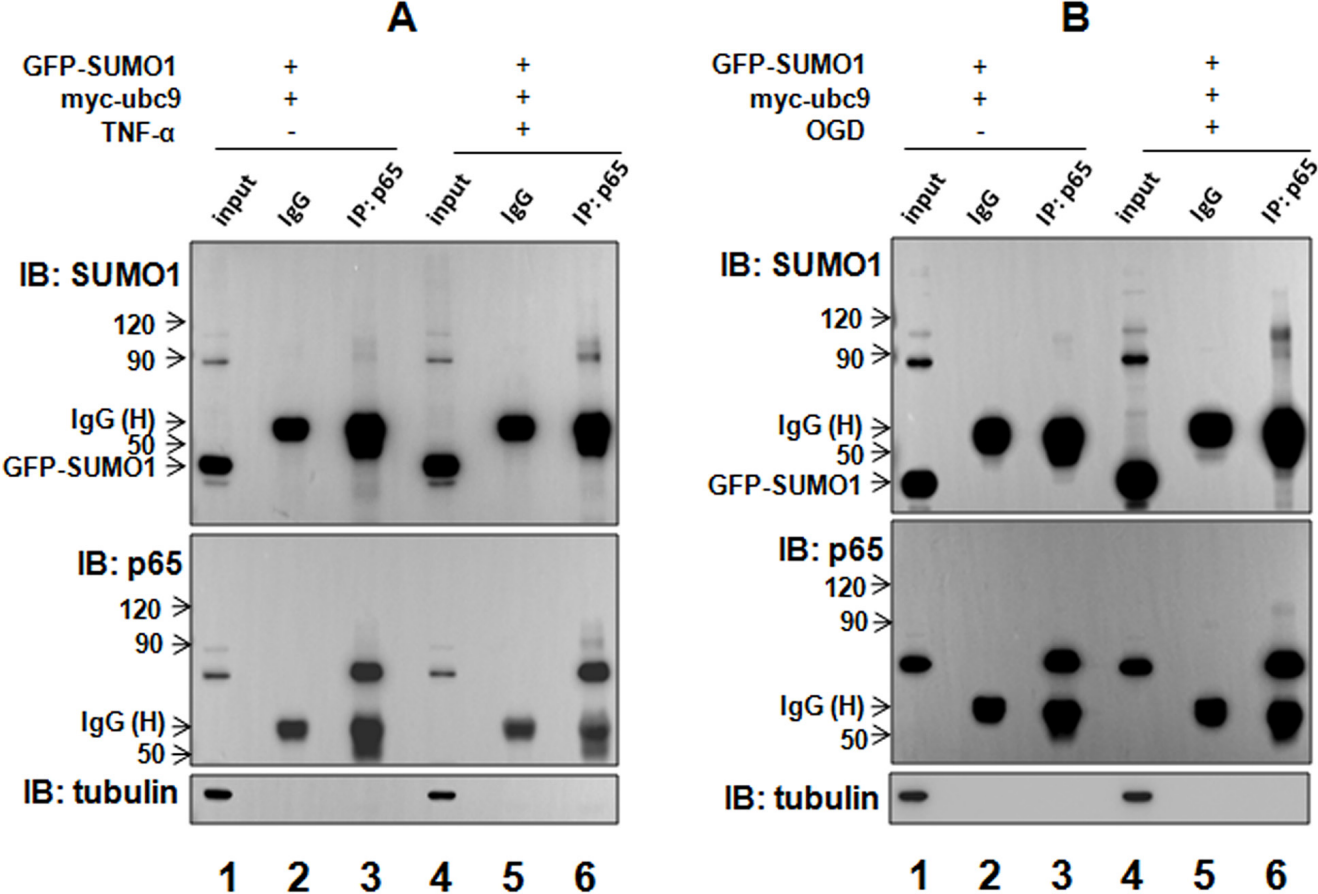
TNF-α and hypoxia enhances the interaction between SUMO1 and p65 SMMC7721 cells were treated with 10 ng/ml TNF-α for 30 min (A) or OGD for 150 min (B) 24 hours after transfection with GFP-SUMO1 and myc-UBC9. The interaction of p65 and SUMO1 was detected with co-immunoprecipitation assay using the anti-p65 antibody. The isotype IgG was used as a negative control, and 2% total lysate was loaded as input.

As mentioned above, TNF-α and OGD treatments up-regulated SUMO1; thus, we sought to determine whether hypoxia and inflammation affect the interaction between SUMO1 and p65. We found that SUMO1-modified p65 SUMOylation was enhanced by TNF-α (Figure 4A, lane 6 vs lane 3) and OGD (Figure 4B, lane 6 vs lane 3). These results suggest that both inflammation and hypoxia enhance the interaction between SUMO1 and p65 and promote SUMO1-modified p65 SUMOylation.

### SUMO1 promotes TNF-α and hypoxia-induced p65 nuclear translocation

**Figure 5:**
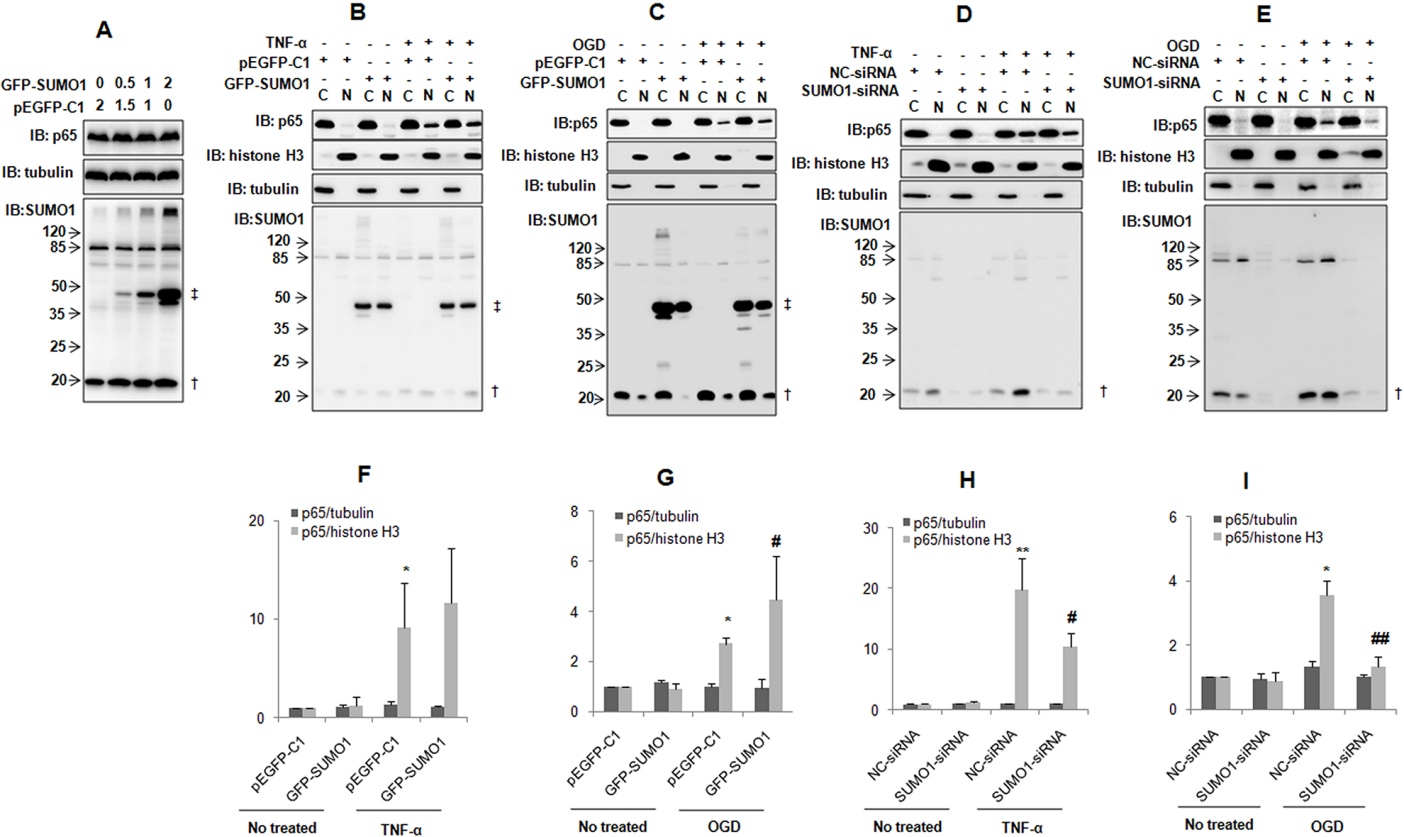
SUMO1 promotes p65 nuclear translocation (**A**) SUMO1 does not affect the p65 protein level. SMMC7721 cells were transfected with different doses of GFP-SUMO1 plasmids. pEGFP-C1 was used to balance the total DNA. The cells were harvested and processed for immunoblotting 24 hours post-transfection. (**B**–**E**) SUMO1 promotes p65 nuclear translocation. SMMC7721 cells were treated with 10 ng/ml TNF-α for 30 min (B and D) or OGD for 150 min (C and E) 48 hours post-transfection with GFP-SUMO1 (B–C) or SUMO1-siRNA (D–E). The nuclear and cytoplasmic proteins were extracted. Tubulin and histone H3 were used as cytoplasmic and nuclear markers. N: nucleus; C: cytoplasm. ^†^: free SUMO1; ^‡^: free GFP-SUMO1. (**F**–**I**) The quantitative data in panels B-E are from three independent experiments. *P < 0.05 and **P < 0.01 compared with the nuclear p65 level of the cells transfected with pEGFP-C1 or NC-siRNA and without any other treatment. ^#^*P* < 0.05 and ^##^*P* < 0.01 compared with the nuclear p65 level of the cells transfected with pEGFP-C1 or NC-siRNA and treated with TNF-α or OGD.

The function of SUMOylation includes the regulation of targeting protein stability and subcellular localization [15, 16]. We verified that p65 can be SUMOylated by SUMO1. Therefore, we further investigated the effect of SUMO1 on p65. We found that SUMO1 over-expression did not affect the p65 protein level (Figure 5A). However, SUMO1 over-expression enhanced hypoxia-induced p65 nuclear import (Figure 5C and 5G). SUMO1 over-expression slightly increased TNF-α-induced p65 nuclear transport (Figure 5B and 5F). In contrast, the knockdown of SUMO1 attenuated hypoxia-induced p65 nuclear translocation (Figure 5E and 5I) and also decreased TNF-α-induced p65 nuclear translocation (Figure 5D and 5H). These results were supported by the immunofluorescence assay results (Figure S2–S3).

### Knockdown of SUMO1 decreases NF-κB transcriptional activity

**Figure 6:**
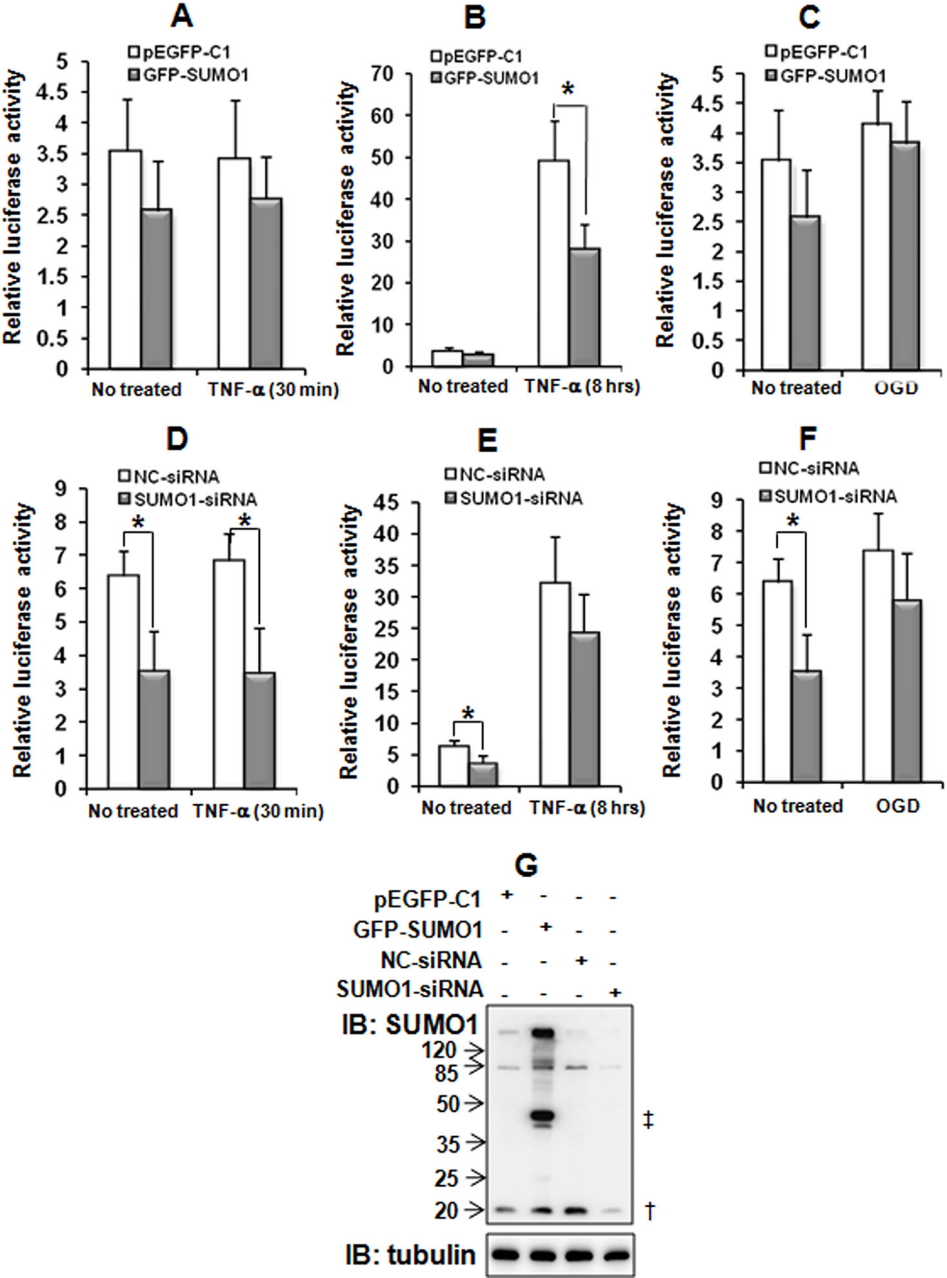
Effects of SUMO1 on NF-κB transcriptional activity SMMC7721 cells were co-transfected with GFP-SUMO1 (**A**–**C**) or SUMO1-siRNA (**D**–**F**) plus κB-luciferase and pRL-TK. The cells were treated with 10 ng/ml TNF-α for 30 min (A and D) or 8 hours (B and E) or with OGD for 150 min (C and F) 48 hours post-transfection. The SUMO1 protein was detected with western blot (**G**) Luciferase assays were performed and the data are presented as the means ± the SDs of three independent experiments. *P < 0.05, compared with the pEGFP-C1 or NC-siRNA control. ^†^: free SUMO1; ^‡^: free GFP-SUMO1.

The results described above indicate that SUMO1 regulates p65 nuclear translocation. Moreover, p65 nuclear translocation activates NF-κB target genes [17]. Therefore, we wondered whether SUMO1 affects NF-κB activation. A luciferase assay revealed that SUMO1 over-expression only reduced NF-κB transcriptional activity under conditions of long-time TNF-α treatment (i.e., after 8 hours but not after 30 min) (Figure 6B). The knockdown of SUMO1 significantly decreased NF-κB activity (Figure 6D–6F), and this effect did not depend on TNF-α or OGD treatment. These findings suggest that endogenous SUMO1 may participate in the nuclear importation of p65, while chronic inflammation-induced SUMO1 may restrict NF-κB over-activation.

### Effects of SUMO1 on the proliferation and migration of hepatoma cells

**Figure 7:**
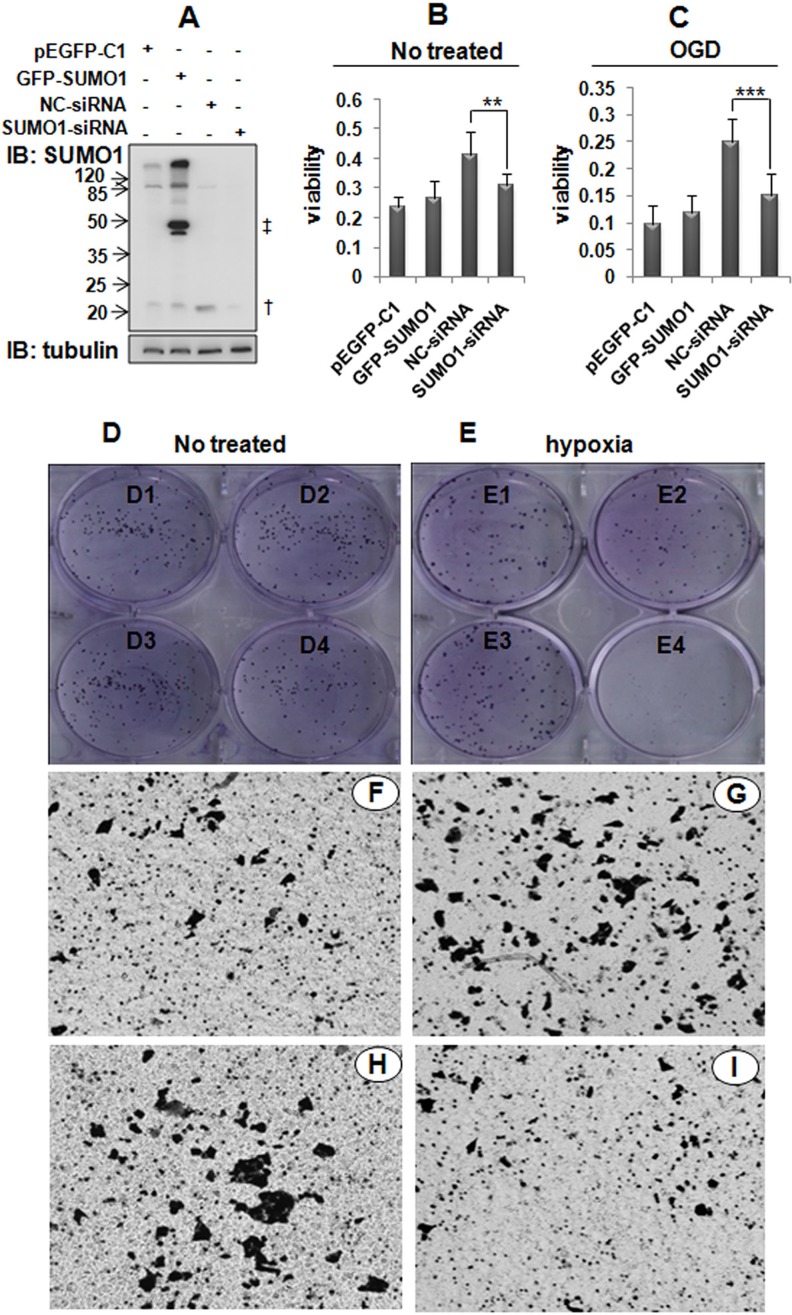
Effects of SUMO1 on the malignant behavior of hepatoma cells (**A**) Detection of SUMO1 expression in SMMC7721 cells using western blot. ^†^: free SUMO1; ^‡^: free GFP-SUMO1. (**B**–**C**) Effects of SUMO1 on hepatoma cell proliferation based on MTT assays. In panel C, the cells were subjected to OGD for 150 min. The data are expressed as the means ± the SDs of at least three independent experiments. **P < 0.01 and ***P < 0.001 compared with NC-siRNA. (**D**–**E**) Effects of SUMO1 on the colony formation of hepatoma cells. SMMC7721 cells were transfected with pEGFP-C1 (**D1** and **E1**), GFP-SUMO1 (**D2** and **E2**), NC-siRNA (**D3** and **E3**), or SUMO1-siRNA (**D4** and **E4**). The cells were subjected to hypoxia (1% O_2_, 5% CO_2_, 94% N_2_) for 48 hours (E). (**F**–**I**) Effects of SUMO1 on the migration of hepatoma cells assessed with transwell assays. The SMMC7721 cells were transfected with pEGFP-C1 (F), GFP-SUMO1 (G), NC-siRNA (H), or SUMO1-siRNA (I).

A number of studies have revealed that p65 is involved in the development of HCC. We demonstrated that the knockdown of SUMO1 decreased p65 nuclear translocation and attenuated NF-κB activity. Therefore, we sought to determine whether SUMO1 affects the malignant biological behavior of hepatoma cells. As illustrated in Figure 7, SUMO1 over-expression promoted the migration (Figure 7F–7G). The knockdown of SUMO1 significantly inhibited proliferation and migration (Figure 7B–7E and 7H–7I), particularly in the hypoxic condition (Figure 7C and 7E). These results suggest that endogenous SUMO1 promotes the development of HCC.

## DISCUSSION

In this study, we report the novel observation that nuclear p65 is significantly correlated with SUMO1 in the liver tissues of HBV-infected HCC patients. We verified that p65 was modified by SUMO1, which was enhanced by both inflammation and hypoxia. Importantly, we have provided evidence that the inhibition of endogenous SUMO1 decreased TNF-α and hypoxia-induced p65 nuclear translocation. Furthermore, our study results revealed that the knockdown of SUMO1 reduced NF-κB transcriptional activity. Additionally, we found that the knockdown of SUMO1 inhibited the proliferation and migration of hepatoma cells.

p65 is one of the most important subunits of NF-κB and primarily localizes to the cytoplasm. p65 nuclear import is a prerequisite for the activation of its target genes and the maintenance of NF-κB transcriptional activity [17, 18]. p65 contains a nuclear localization signal (NLS) domain that promotes the nuclear enrichment of p65 [19]. However, dual-luciferase assays have revealed that NLS-mutated p65 retains strong transcriptional activity compared with the vector, which suggests that p65 may be imported into the nucleus independently of its NLS [20]. A recent study demonstrated that the RanGDP/AR pathway, which is NLS-independent, can regulate p65 nuclear translocation [21]. RanGDP is converted from RanGTP by RanGAP1 at the cytoplasmic side of the nuclear pore complex (NPC) where RanGAP1 is SUMOylated by SUMO1 [22, 23]. Therefore, we speculate that SUMO1-related SUMOylation might be associated with p65 nuclear translocation.

SUMOylation was first noted in the NPC, where it regulates the interaction of RanGAP1 and RanBP2. RanGAP1 and RanBP2 contribute to the nuclear import of proteins [23, 24]. Recently, the RanBP2/RanGAP1 * SUMO1/UBC9 complex was reported to be a multisubunit SUMO E3 ligase in the NPC that regulates the nuclear import of SUMOylated target proteins [25, 26]. Accordingly, SUMO1 promotes the nuclear translocation of target proteins, such as SAE2, ZIC3, and ErbB4 [27–29]. Additionally, SUMO1 promotes the nuclear translocation of IGF-1R, which lacks a NLS domain [30]. Because SUMO1 is primarily localized in the nuclear membrane [31], the protein levels of SUMO1 in the cytoplasm and nuclei vary when western blot assays are performed following the extraction of nuclear and cytoplasmic proteins [32, 33]. This study was aimed to observe whether SUMO1 increases p65 nuclear localization under TNF-α and OGD treatment. To avoid cytoplasm contamination, we washed the nuclear pellet for more than three times during nucleus/cytoplasm isolation. Therefore, nuclear SUMO1 might be lost during nucleus/cytoplasm isolation. However, nuclear p65 was still increased after SUMO1 over-expression under OGD treatment. These results clearly demonstrated that SUOM1 promotes p65 nuclear localization under OGD condition. Our results revealed that SUMO1 and nuclear p65 were increased in the tumor tissues of HCC patients, and these findings are in accordance with the results recently reported by Uhlen et al. [34]. Our findings suggest a close relationship between SUMO1 and nuclear p65 in liver tissues (R = 0.851, *P* = 0.002). We further verified that p65 was SUMOylated by SUMO1, and the increase in endogenous SUMO1 enhanced p65 nuclear translocation. However, SUMO2 over-expression inhibits p65 nuclear import in dendritic cells [35]. Additionally, our recent work demonstrated that SUMO2/3 over-expression promotes the stability of cytoplasmic p65 [12]. These findings suggest that SUMO1, and not SUMO2/3, promotes p65 nuclear import.

The nuclear import of p65 is associated with NF-κB activation. Therefore, we investigated whether SUMO1 affects NF-κB activation. Our results indicated that both SUMO1 over-expression and the knockdown attenuated NF-κB activation. These findings seem contradictory; however, the effects of SUMO1 over-expression and knockdown on NF-κB activation are different (Figure 6). The effects of the inhibition of SUMO1 over-expression on NF-κB transcriptional activity were dependent on long-time TNF-α stimulation, whereas SUMO1 knockdown suppressed NF-κB transcriptional activity independently of inflammatory stimulation. These findings suggest that endogenous SUMO1 may promote p65 nuclear import, while chronic inflammation-induced SUMO1 may restrict NF-κB over-activation. A previous study demonstrated that SUMO1 protein is mainly localized to the nuclear membrane [31]. We speculate that the knockdown of SUMO1 may attenuate the formation of the RanBP2/RanGAP1 * SUMO1/UBC9 complex in the NPC and affect p65 nuclear translocation. However, in addition to the nuclear membrane, SUMO1 protein is found throughout the cell [31] and participates in protein SUMOylation [36]. A number of studies have demonstrated that the SUMO1-mediated SUMOylation of different members of the NF-κB pathway exerts different effects on the NF-κB activity. For example, another NF-κB subunit, the RelB protein, exhibits increased transcriptional activity compared with the RelB-SUMO1 fusion protein. Moreover, the level of SUMO1-related RelB-SUMOylation is much higher than that of p65-SUMOylation after cells are transfected with the SUMO1 plasmid [37], which may explain why SUMO1 over-expression decreased NF-κB activity. Moreover, one of the RelB target genes is IκBα [38]. Therefore, these results demonstrated that RelB-SUMO1 can decrease itself transcriptional activity and IκBα mRNA levels, which might be the reason that SUMO1 over-expression decreased NF-κB activation and increased p65 nuclear import. A number of studies have revealed that p65 nuclear translocation promotes the development of HCC [39–41]. We found that SUMO1 over-expression increased p65 nuclear translocation and promoted the proliferation and migration of hepatoma cells. Similarly, the knockdown of SUMO1 inhibited p65 nuclear import and suppressed the proliferation and migration of hepatoma cells. These findings support the notion that p65 nuclear import promotes HCC development.

Because the liver samples were collected from HBV-infected HCC patients, the tissues might have suffered from long periods of inflammatory responses and hypoxia [42, 43]. Additional research has demonstrated that hypoxia increases SUMO1 levels *in vitro* in T84 cells and *in vivo* in mouse brains and hearts [44, 45]. We recently reported that short-term inflammatory stimulation up-regulates cytoplasmic SUMO2/3 protein expression [12]. However, long-term inflammation increases SUMO1 protein expression. Moreover, inflammation and hypoxia enhanced SUMO1-modified p65 SUMOylation in the liver tissues and hepatoma cells. Some studies have demonstrated that hypoxia and inflammation enhanced p65 nuclear translocation and increase NF-κB activation [46, 47], and our findings confirmed these results. Our study also revealed that SUMO1 knockdown inhibits the malignant behavior of hepatoma cells, which accords with the finding that SUMO1-siRNA inhibits the growth of SMMC7721 cells [3], especially after the cells are exposed to hypoxia.

**Figure 8:**
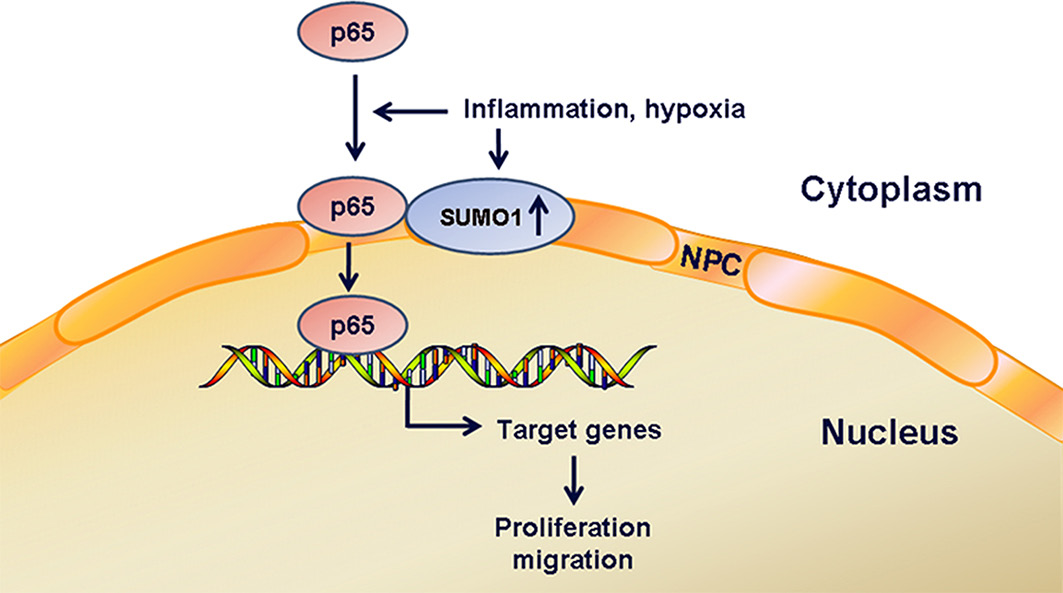
Schematic diagram Hypoxia and inflammation increase SUMO1 protein levels and the interaction of SUMO1 and p65, which promotes p65 nuclear transport. Consequently, the proliferation and migration of hepatoma cells are promoted.

Taken together, these findings indicate that short-term inflammatory stimulation increases the cytoplasmic SUMO2/3 level, which leads to cytoplasmic enrichment of p65 in hepatoma cells. Hypoxia and long-term inflammation result in the increases in SUMO1 protein levels. Moreover, inflammation and hypoxia enhance SUMO1-modified p65 SUMOylation, which may be involved in the promotion of p65 nuclear transport. Consequently, the proliferation and migration of hepatoma cells were promoted (Figure 8). Our study might aid the understanding of the mechanisms of hepatocarcinogenesis and development.

## MATERIALS AND METHODS

### Cells and human liver specimens

HepG2 cells were purchased from CHI Scientific (Jiangyin, China). SMMC7721 cells were obtained from Cobioer Biosciences (Nanjing, China). The human liver tissues were obtained from the First Affiliated Hospital of Anhui Medical University and the Chinese People's Liberation Army 123 Hospital. The use of human liver tissues adhered to the tenets of the Declaration of Helsinki. This study was approved by the Ethics Committee of Anhui Medical University (No. 20131359).

### Plasmids

The GFP-SUMO1 plasmid was a kind gift from Prof. Steve Jackson (University of Cambridge, UK). The Myc-Ubc9 construct was a kind gift from Prof. Hiderou Yoshida (University of Hyogo, Japan). The 3 × κB-Luc and pRL-TK plasmids were kind gifts from Prof. Wancheng Li (University of Nebraska Medical Center, USA).

### Reagents and antibodies

Antibodies against tubulin (Sigma-Aldrich, St. Louis, MO, USA), histone H3 and SUMO1 (Abcam, Cambridge, MA, USA), p65 (Millipore, Darmstadt, Germany), rabbit IgG (Cell Signaling Technology, Beverly, MA, USA), Alexa Fluor 488-conjugated and 568-conjugated IgG (Invitrogen, *Carls*-bad, CA, USA) were used. Lipofectamine 2000 and Opti-MEM were obtained from Invitrogen (*Carls*-bad, CA, USA). N-Ethylmaleimide (NEM) was purchase from Sigma-Aldrich (St. Louis, MO, USA). TNF-α was obtained from R & D Systems (Minneapolis, MN, USA). Pierce Protein A agarose was obtained from Thermo (Rockford, IL61001, USA). The dual-luciferase reporter assay kit was obtained from Promega (Madison, WI, USA).

### Immunohistochemistry and correlation analysis

Sections of human liver tissue from 5 hepatitis patients and 8 HCC patients were deparaffinized in dimethylbenzene and rehydrated through graded ethanol. The antigens were retrieved, and the endogenous peroxidase activity was quenched. Next, the sections were incubated with goat serum and the corresponding antibodies. The sections were stained with 3, 3′-diaminobenzidine tetrahydrochloride (DAB), and the nuclei were counterstained with hematoxylin. The positive cells were counted in six independent high-magnification (×400) fields per section, and the average was calculated. The percentage of positive cells was analyzed as the ratio of the positive cells to the total cells.

### Immunofluorescence staining

After deparaffinization and antigen retrieval, the liver sections were incubated with goat serum, primary antibodies and immunofluorescence-labeled second antibodies to perform the dual fluorescence staining. Meanwhile, the nuclei were detected with 4′, 6-diamidino-2-phenylindole (DAPI), and the images were collected with fluorescence microscopy.

### Immunocytochemistry

The cells on the coverslips were fixed with paraformaldehyde, permeabilized in Triton X-100, and blocked with 5% BSA. After incubation with the corresponding antibodies, the cells were stained with DAPI.

### OGD

The **c**ells were cultured in glucose-free balanced salt solution (BSS: 116 mM NaCl, 14.7 mM NaHCO_3_, 5.4 mM KCl, 1.8 mM CaCl_2_, 1 mM NaH_2_PO_4_, 0.8 mM MgSO_4_, and 10 mM HEPES, pH 7.4) rather than DMEM and fetal bovine serum [48], and were then exposed to an anoxic gas mixture (94% N_2_, 5% CO_2,_ 1% O_2_) for 150 minutes using an anoxic chamber (Eppendorf company Galaxy 170R).

### Co-immunoprecipitation

The following procedures were performed at 4^°^C or on ice. Pierce Protein A agarose and p65 antibody were incubated in an end-over-end mixer for 90 min. The isotype IgG antibody was used as a negative control. The tissue lysates or whole cell lysates was prepared in a lysis buffer (50 mM HEPES, 150 mM NaCl, 2 mM EGTA and 0.1% Triton X-100, pH 7.4) containing 20 mM NEM and Complete^™^ protease inhibitors to prevent p65 de-SUMOylation and protein degradation. The lysates were centrifuged, and the supernatants were mixed with Pierce Protein A agarose for 4 hours. The Pierce Protein A agarose was then eluted, and the bound proteins were analyzed using western blot.

### Dual-luciferase reporter assay

SMMC7721 cells were co-transfected with pRL-TK, 3 × κB-luc and GFP-SUMO1 or SUMO1-siRNA. pEGFP-C1 and NC-siRNA were used as controls. Forty-eight hours post-transfection, the cells were treated with or without TNF-α (10 ng/ml) for different durations (30 min and 8 hours) or OGD for 150 min. NF-κB activity was measured with the dual-luciferase reporter assay system. The experiments were repeated three times.

### Proliferation assay

SMMC7721 cells were transfected with GFP-SUMO1 or SUMO1-siRNA or the corresponding controls. Forty-eight hours post-transfection, the viability of the cells was quantified with a MTT assay. Meanwhile, 200 cells were seeded in each of the wells of 6-well plates for the colony-formation assay, and the colonies were stained 12 days later.

### Transwell assay

SMMC7721 cells were transfected with GFP-SUMO1, SUMO1-siRNA or the corresponding controls. Twenty-four hours post-transfection, 4 × 10^4^ cells were planted in the upper compartment for the transwell assay. Twenty-four hours later, the migrated cells on the lower membrane were stained, and the images were observed under a microscope.

### Statistical analysis

The quantitative data are presented as the means ± the SDs. The Kolmogorov-Smirnov test was used to analyze the data distributions. Paired-samples *T* tests were used to compare the means of the positive cell percentages of the tumors and corresponding non-tumor liver tissues from the same patient. The means of pairs of groups (i.e., pEGFP-C1 vs GFP-SUMO1 and NC-siRNA vs SUMO1-siRNA) were compared with independent-sample *T* tests. Because the parameters were normally distributed, Pearson's correlation tests were used to assess the correlations of the percentages of SUMO1-positive cells with the percentages of nuclear p65-positive cells. *P* < 0.05 was considered statistically significant.

## SUPPLEMENTARY MATERIALS FIGURES


