# Supplementary material for: Small ubiquitin-related modifier 1 is involved in hepatocellular carcinoma progression via mediating p65 nuclear translocation
Source: Oncotarget. 2016 Mar 14;7(16):22206–18. doi: 10.18632/oncotarget.8066 (PMC5008356; doi:10.18632/oncotarget.8066)
Supplement: Supplementary file 1 [file oncotarget-07-22206-s001.pdf]

Small ubiquitin-related modifier 1 is involved in hepatocellular carcinoma progression via mediating p65 nuclear translocation

Supplementary Materials

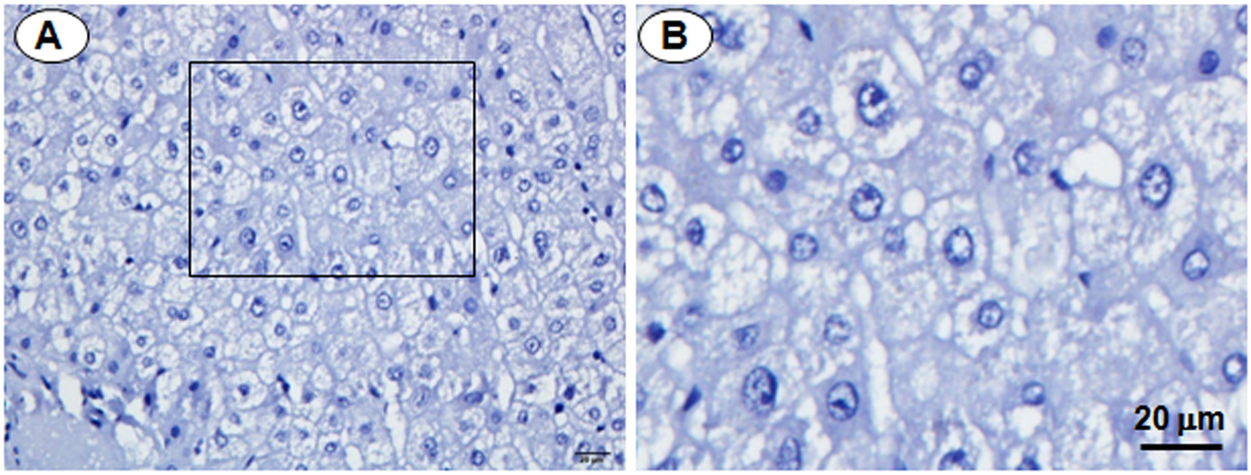

Supplementary Figure S1: Negative control for the immunohistochemistry assay. Isotype IgG was used to replace the primary antibody in the immunohistochemistry assay. The rectangles in (**A**) is magnified in (**B**). Scale bar = 20 µm.

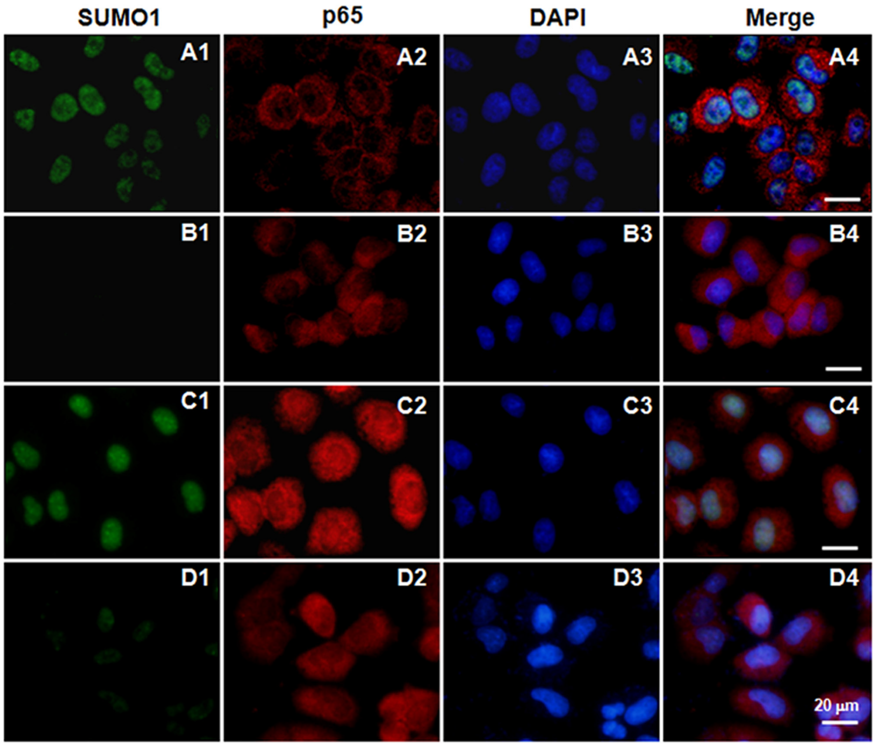

Supplementary Figure S2: Knockdown of endogenous SUMO1 attenuates TNF-α-induced p65 expression. SMMC7721 cells were transfected with NC-siRNA (**A** and **C**) or SUMO1-siRNA (**B** and **D**). Forty-eight hrs later, the cells were treated with TNF-α (10 ng/ml) for 30 min (C and D) or vehicle control (A and B). Subsequently, the cells were double labeled using immunofluorescent staining with the antibodies against SUMO1 (green) and p65 (red). The nuclei were stained with DAPI (blue). Scale bar = 20 µm.

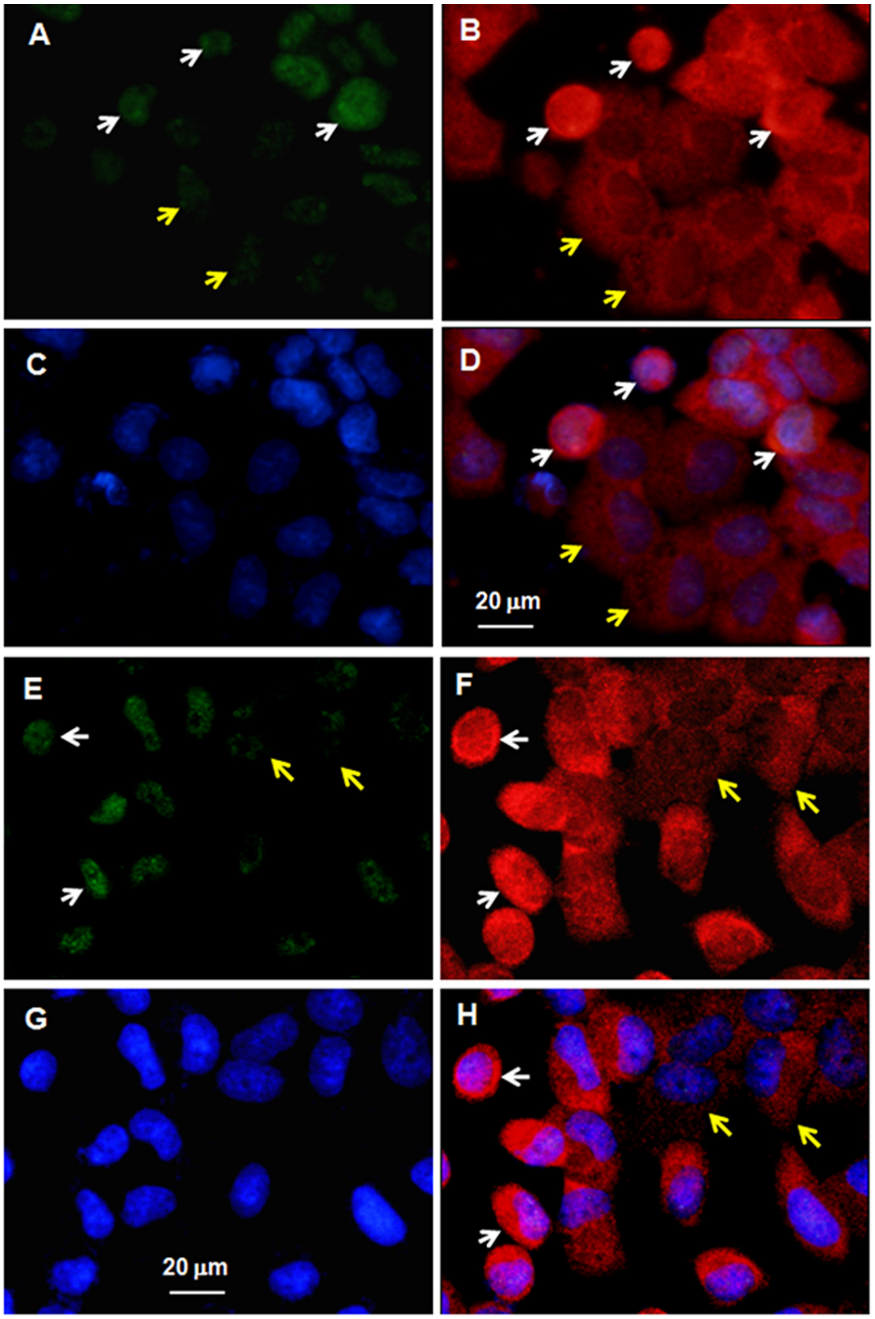

Supplementary Figure S3: Knockdown of endogenous SUMO1 attenuates TNF-α and hypoxia-induced p65 nuclear translocation. SMMC7721 cells were transfected with SUMO1-siRNA. Forty-eight hours later, the cells were treated with TNF-α (10 ng/ml) for 30 min (**A–D**) or with OGD (**E–H**). Subsequently, the cells were double labeled using immunofluorescent staining with the antibodies against SUMO1 (green) and p65 (red). The nuclei were stained with DAPI (blue). Scale bar = 20 μm. The white arrows indicate cells expressing SUMO1 in the nuclei. The yellow arrows indicate cells expressing very low levels of SUMO1 in the nuclei.
